# Validation of the phenol red thread test in a Chinese population

**DOI:** 10.1186/s12886-023-03250-3

**Published:** 2023-12-07

**Authors:** Yiran Hao, Tao Jin, Lei Zhu, Mengnan Zhao, Silu Wang, Zhongying Li, Lei Tian, Ying Jie

**Affiliations:** 1 Beijing Institute of Ophthalmology, Beijing Tongren Eye Center, Beijing Tongren Hospital, Capital Medical University, Beijing Ophthalmology and Visual Sciences Key Laboratory, Beijing, 100730 China; 2 Beijing Advanced Innovation Center for Big Data-Based Precision Medicine, Beihang University and Capital Medical University, Beijing, China

**Keywords:** Phenol red thread test, Dry eye, Tear volume, Repeatability, Reproducibility

## Abstract

**Background:**

To investigate the validation of phenol red thread (PRT) test in a Chinese population by evaluating the intraobserver repeatability and interobserver reproducibility, determining correlations between the PRT test and other dry eye disease (DED) parameters including tear meniscus height (TMH) and Schirmer I test, and testing the accuracy of diagnosing DED when using the PRT test alone.

**Methods:**

A total of 108 eyes were involved in this prospective and diagnostic study, and were divided into two groups (with and without DED). Each subject underwent a series of ocular surface examinations, including Ocular Surface Disease Index (OSDI) questionnaire, non-invasive tear breakup time (NIBUT), tear meniscus height (TMH) assessment, PRT test, fluorescein tear breakup time (FBUT), corneal fluorescein staining and Schirmer I test.

**Results:**

In the experimental group and the control group, the intra-class correlation coefficients (ICCs) of the repeatability were 0.747 and 0.723, respectively (all *P* < 0.05). The ICCs of the reproducibility in both groups were 0.588 and 0.610, respectively (all *P* < 0.05). The PRT test correlated weakly with the Schirmer I test and the tear meniscus height, with Spearman coefficients of 0.385 and 0.306, respectively (all *P* < 0.05). The PRT test is available to diagnose DED, with an area under the curve of 0.806 and a Youden index of 0.556 at the cutoff point of 8.83 mm.

**Conclusions:**

The PRT test can provide patients a comfortable, timesaving and less irritating approach to screening and diagnosing DED compared to Schirmer I test.

## Introduction

A stabilized preocular tear film is of significant importance to ocular health as it plays an essential role in the protection and moisturization of the cornea and represents the front of the visual system where the light first enters and refracts. Not only can the tear film nourish the cornea, but it can also prevent the cornea from incurring injury or infection. However, many factors can break the balance of the tear film and lead to the loss of tear production, tear quality and tear supplementation, thus contributing to dry eye disorders, including the use of contact lenses, an irregular circadian clock and systemic diseases [1–3]. According to the TFO DEWS II report in 2017, there was no single “gold standard” symptom or sign that completely correlates with dry eye diseases (DED) [4]. So, it is necessary to find out an effective and accurate method to evaluate the homeostasis of tear film and diagnose DED.

The loss of tear volume has been considered as one of the main characteristics of DED [4]. Total tear volume is predominantly decided by secretions from the lacrimal glands along with a small secretion arising from the accessory lacrimal glands in the conjunctiva [5]. According to Craig et al*.*, tears can be classified into four types: basal, reflex, emotional and closed-eye [6]. Basal tears, mainly produced by the lacrimal gland, form the constant tear film, and cover the ocular surface most of the time; deficiencies may result in DED [5]. The approach to measure the volume of basal tears seems to be an essential way of diagnosing DED.

Several methods have been invented to measure tear secretion and tear volume, such as the Schirmer test, the phenol red thread (PRT) test, and tear meniscus height (TMH) assessment. The phenol red thread test, also known as the Hamano test, was introduced by Hamano et al*.* in 1983 as a replacement for the Schirmer test [7]. In this technique, a more sensitive thread is dipped with phenol red and used to measure tear volume in the inferior conjunctival sac. This test is much more comfortable for the patient than the Schirmer test strip as it is softer, thinner and only takes a few seconds to conduct; consequently, reflex tears are reduced and the test is more accurate with regards to determining the constant tear volume.

As the PRT test is more comfortable, timesaving and less irritating for the patient, it would be warmly welcomed clinically once it has been verified and validated, especially in busy outpatient services. Therefore, this study aimed to investigate the repeatability and reproducibility of the PRT test in both DED patients and normal people from China, which would be more persuasive and practical clinically. And the study also purposed to determine how the PRT test correlates with the Schirmer test and TMH assessment, and assess the accuracy of the PRT when used to diagnose DED using current diagnostic criteria, so as to raise an effective and accurate method of diagnosing DED. Collectively, these findings can be used as evidence to support clinical translation.

## Methods

### Subject recruitment

A prospective and diagnostic study recruited a total of 108 eyes from 63 patients with ocular disorders who visited the Outpatient Department of Beijing Tongren Hospital, Beijing, China between February and April 2022. The subjects were divided into two groups according to DED diagnostic criteria [8]. The experimental group included 63 eyes with DED while the control group included 45 eyes without DED.

The diagnosis of DED was made according to the consensus on DED in China (2020) and required at least one of the following symptoms: dryness, sandiness, burning, tiredness, discomfort, redness, and blurred vision with an Ocular Surface Disease Index (OSDI) questionnaire score ≥ 13 scores [8]. In the case of the above symptoms, the following signs were necessary for diagnosis: (1) fluorescein tear film breakup time (FBUT) ≤ 5 s or a non-anesthesia Schirmer Ι test value ≤ 5 mm/5 min; or (2) 5 s < FBUT ≤ 10 s or 5 mm/5 min < non-anesthesia Schirmer Ι test ≤ 10 mm/5 min, accompanied by a corneal fluorescein staining positive(≥ 5 dots) [8].

All included subjects signed informed consents forms in accordance with the tenets of the Declaration of Helsinki. The study was approved by the Institutional Review Board of Beijing Tongren Hospital, Beijing, China. The subjects over 18 years-of-age with the same ethnicity (Chinese) and were willing to participate were included in the study. Patients who had previously undergone corneal or ocular surgery, had worn contact lenses in the previous 24 h, had any history of Stevens Johnsons Syndrome or Sjögren syndrome, or other systemic illness or risk factors known to impact the tear film, or had undergone any treatment related to dry eyes within the previous two weeks, or those who had taken medication recently were excluded.

### Ocular examinations

Each subject was required to carry out specific ocular examinations in the following order:

- 1. Ocular Surface Disease Index (OSDI) questionnaire
- 2. Non-invasive tear breakup time (NIBUT)
- 3. Tear meniscus height (TMH) assessment
- 4. Phenol red thread (PRT) test (in triplicate)
- 5. Fluorescein tear breakup time (FBUT)
- 6. Corneal fluorescein staining
- 7. Schirmer I test

Subjects rested for at least 10 min before the NIBUT, TMH assessment, FBUT and Corneal fluorescein staining. A 15-min rest was arranged before the PRT test and the Schirmer I test [9, 10]. This practice ensured that the tear film, tear volume and ocular condition recovered to its original status. All examiners and analyzers were blinded from the subjects clinical and demographic details, and all assessments were performed in a dimly lit room (temperature 20–25 °C, humidity 30–40%) between 8 am and 4 pm on a single day.

#### Dry eye symptoms questionnaire

Each subject was asked to complete the OSDI questionnaire (validated Chinese version [11]). This questionnaire featured three segments, including ocular symptoms, vision-related function, and environmental triggers, to evaluate subjective dry eye symptoms in a detailed manner [12]; the outcome ranges from 0 to 100 with higher scores referring to severer symptoms.

#### Non-invasive tear breakup time (NIBUT)

The NIBUT was tested with an Oculus Keratograph (K5M; Oculus Optikgeräte GmbH, Wetzlar, Germany). Subjects rested their chin on the chin rest with their foreheads pressed against the forehead band, watched the fixation pattern inside the device and blinked according to instruction. The tear breakup time was examined automatically and the first and average tear breakup time was reported [13].

#### Tear meniscus height (TMH) assessment

The Oculus Keratograph was also used for TMH assessment. Subjects placed their chin and forehead as discussed before and watched a fixed pattern without blinking. An image of the tear meniscus was captured by the tester, and the height of the tear meniscus was measured by the integrated ruler at the central point perpendicular to the lid margin relative to the pupil center. The TMH was measured by Keratograph 5 M for consecutive 3 times, and the mean value was recorded [13].

#### Phenol red thread (PRT) test

A yellow cotton thread soaked with phenol red (Tianjin Jingming New Technological Development Co., Ltd) was used for this test. The thread, 70 mm in length with a 3 mm fold at the beginning, was fixed in the strip with a scale (10 mm in width and 70 mm in length) and covered with plastic film. The examiner removed the front of the plastic film without touching the thread, inserted the folded portion into the middle and outer 1/3 of the inferior palpebral conjunctiva and started timing. The test was conducted without topical anesthesia. When the tears are absorbed, the thread turns from white to orange, and then to red. After 20 s, the thread was pulled out and the length of the thread stained red was measured. The length of the color-changing thread refers to the volume of tears [7].

The test was conducted three times for each subject. Two consecutive tests were performed by the same examiner with a 15-min interval to measure intra-observer repeatability. Then, another examiner carried out an individual test with another 15-min interval to test the inter-examiner reproducibility.

#### Fluorescein tear breakup time (FBUT)

Before conducting the test, an aseptic fluorescein strip moisturized with normal saline was dipped into the subject’s conjunctival sac in both eyes. The subject was then required to blink several times to ensure that the tear film and cornea were stained evenly. Then, the examiner observed the tear film through a cobalt-blue filter with a slit-lamp microscope with the subject’s eyes opened. The interval between the last complete blink and the appearance of the first corneal black spot in the stained tear film was recorded three times by a stopwatch and the mean value was calculated as the FBUT. Records that were less than or equal to 5 s were regarded as abnormal.

#### Corneal fluorescein staining

As the cornea has been stained during the FBUT test, the examiner could observe the cornea through a cobalt-blue filter using a slit-lamp microscope directly. The number of stained spots was counted and the appearance of stained spots was considered abnormal [14].

#### Schirmer I test

Standardized Schirmer I test strips were utilized to assess aqueous tear production. The strips were placed without anesthesia over the middle and outer 1/3 of the inferior palpebral conjunctiva in both eyes simultaneously and then left for 5 min with the eyes closed. The length of the strip that had become wet was read as the outcome and a reading less than or equal to 5 mm was considered abnormal [15].

### Statistical analysis

As for a study design with 2 repeated measures, the uncertainty was set to be 20% in the repeatability and reproducibility result, which means the sample size for precision studies must be over 48 according to the formula [16]:

$$1.96\frac{Sw}{\sqrt{2n\left({n}^{\prime}-1\right)}}=0.2Sw$$

Sw: within-subject standard deviation; n: number of the subject; n’: number of repeated measurements.

As for the study designed to evaluate the diagnostic validation of the PRT test, the sensitivity was set to be 90% and the allowable error was set to be 10% according to the previous study [17, 18], which means the sample size for precision studies must be over 84 according to the formula:

$$n=\frac{Z_{1-\beta} \sqrt{\pi\left(1-\pi\right)}+Z_{1-\alpha}\sqrt{\left(\pi -\delta\right)\left(1-\pi +\delta\right)}}{\delta ^{2}}$$

π: sensitivity; σ: permissible fluctuation range of sensitivity; β: value of Class II error is 0.8, and Z (1-β) is 0.84; α: value of Class I error is 0.05, and Z (1-α) is 1.65

SPSS version 26.0 (SPSS, Inc., Chicago, IL, USA) was used to conduct statistical analysis. The Shapiro–Wilk test and histogram were used to test the raw data for normality. The independent-sample t test and Kruskal–Wallis H test was used to assess the differences between experimental group and control group. The intra-class correlation coefficient (ICC) and Bland–Altman analysis were carried out to assess the intra-observer repeatability and the inter-examiner reproducibility. The Spearman rank-order correlation coefficient was calculated to evaluate correlations among the PRT test and other ocular examinations. Receiver operating characteristic (ROC) curve analysis was conducted to assess the diagnostic accuracy of the PRT test. The standard deviation (SD) and coefficient of variance (CV) were computed to assess the fluctuation of certain ocular examination parameters. All *P* values were two-sided and were considered statistically significant at *P* < 0.05.

## Results

### Demographics

A total of 108 eyes (from 63 patients) were recruited for the study; 63 eyes with DED were included in the experimental group while 45 eyes without DED were included in the control group. Table 1 shows the statistical characteristics of the subjects, while Table 2 shows the mean values and coefficients of variance for the PRT tests, TMH assessment, and Schirmer I test. Independent-sample t test and Kruskal–Wallis H test were used to assess the differences between experimental group and control group. Significant differences were found between experimental group and control group of the ocular parameters including ODSI, PRT test-first, PRT test-average, TMH and Schirmer I test (all *P* < 0.05).

**Table 1:** Statistical characteristics of the subjects

Parameters	Experimental Group	Control Group
*n* (eyes)	63	45
Age (years)	27.00 (23.00,31.00)	34.00 (22.00,29.00)
Gender (Male/Female)	5/58	5/40
OSDI (score)	20.00 (10.42,22.92)	10.42 (2.64,22.50)
PRT test-first (mm)	7.04 ± 3.01	10.33 ± 3.55
PRT test-average (mm)	7.07 ± 2.61	10.27 ± 2.88
TMH (mm)	0.189 ± 0.053	0.219 ± 0.05
Schirmer I test (mm)	4.00 (1.00,6.00)	16.00 (6.75,30.00)

**Table 2:** Mean values and coefficients of variance for the PRT tests, TMH assessment, and Schirmer I test

Parameters	n (eyes)	Mean ± SD^a^/ Median(P25,P75)	CV^b^ (%)
PRT test-first (mm)	108	8.00 (5.00,11.00)	43.04
PRT test-second (mm)	108	8.00 (6.00,11.00)	43.31
PRT test-third (mm)	108	8.00 (6.00,10.00)	39.36
PRT test-average (mm)	108	8.40 ± 3.14	37.38
TMH (mm)	108	0.201 ± 0.054	26.87
Schirmer I test (mm)	108	6.00 (3.00,16.00)	101.06

^a^*SD* standard deviation, ^b^*CV* coefficient of variance

### Intra-observer repeatability and inter-observer reproducibility for the PRT test

**Fig. 1:**
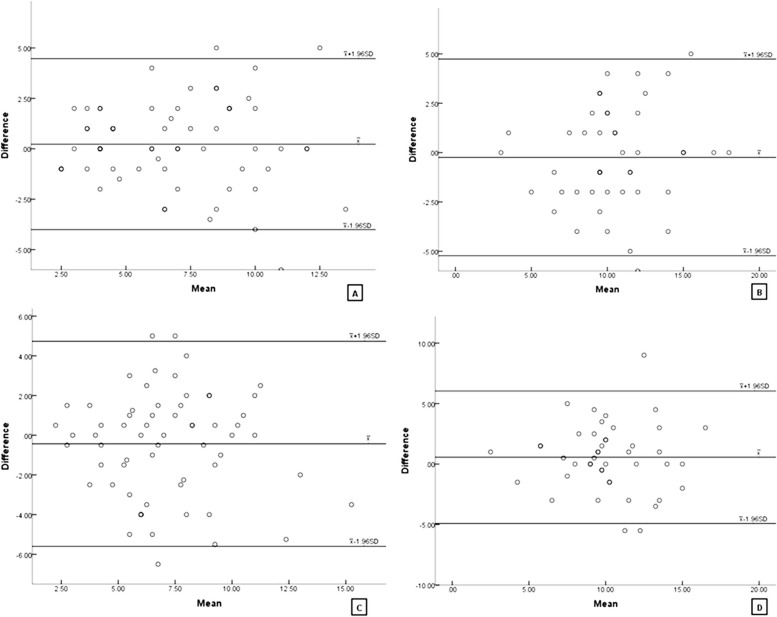
Bland–Altman analysis diagrams for the intra-observer repeatability test and the inter-observer reproducibility test for the two groups. The difference between the measurements (Difference) is plotted on the vertical axis and their mean is plotted on the horizontal axis (Mean). Despite the zero line, the middle horizontal dotted line represents the mean difference and the two horizontal lines, one above and the other below, represent the 95% limits of agreement. **A** Intra-observer repeatability for experimental group. **B** Intra-observer repeatability for control group. **C** Inter-observer reproducibility for experimental group. **D** Interobserver reproducibility for control group

ICC data, 95% confidence intervals for the ICC, and Bland–Altman 95% limits of agreement for the PRT test are shown in Table 3. Figure 1 shows Bland–Altman analysis diagrams for the intra-observer repeatability test and the inter-examiner reproducibility test for the two groups.

**Table 3:** Intra-observer repeatability and inter-observer reproducibility for the PRT^a^ test

	Intra-observer repeatability		Inter-observer reproducibility	
	Experimental group	control group	Experimental group	Control group
*n*	63	45	63	45
ICC^b^	0.747	0.723	0.588	0.610
95% CI^c^ for ICC	0.614 to 0.761	0.548 to 0.838	0.401 to 0.728	0.391 to 0.764
*P* value	< 0.001	< 0.001	< 0.001	< 0.001
Bland–Altman 95% limits of agreement	-4.008 to 4.468	-5.227 to 4.738	-5.597 to 4.732	-4.908 to 6.041

^a^*PRT* phenol re thread, ^b^*ICC* intraclass correlation coefficient, *95%* ^c^*CI* 95% confidence interval for the mean

The intra-observer repeatability test was conducted by the same examiner over two consecutive tests, the ICC values were 0.747 for the experimental group and 0.723 for the control group (both *P* < 0.001). The Bland–Altman 95% limits of agreement for the experimental group and control group were -4.008 to 4.468 and -5.227 to 4.738, respectively. According to the Bland–Altman analysis diagrams, 6.35% and 4.44% of datapoints fell outside the 95% limits of agreement. The ICC values in both groups were over 0.7 with *P* < 0.05; the number of datapoints falling outside of the Bland–Altman 95% limits of agreement were < 10%, thus indicating ‘good’ intra-observer repeatability.

For the inter-observer reproducibility test, the second test was carried out by another examiner. The ICC values for the experimental group and the control group were 0.588 and 0.610, respectively (both *P* < 0.001). The Bland–Altman 95% limits of agreement for were -5.597 to 4.732 and -4.908 to 6.041, respectively. According to the Bland–Altman analysis diagrams, the proportion of datapoints that fell outside the 95% limits of agreement were 4.76% and 6.67%, respectively. The ICC values in both groups were over 0.6 with *P* < 0.05, and the datapoints that fell outside the Bland–Altman 95% limits of agreement were < 10%, thus indicating ‘general’ interobserver reproducibility.

### Correlations between the PRT test and DED parameters

**Fig. 2:**
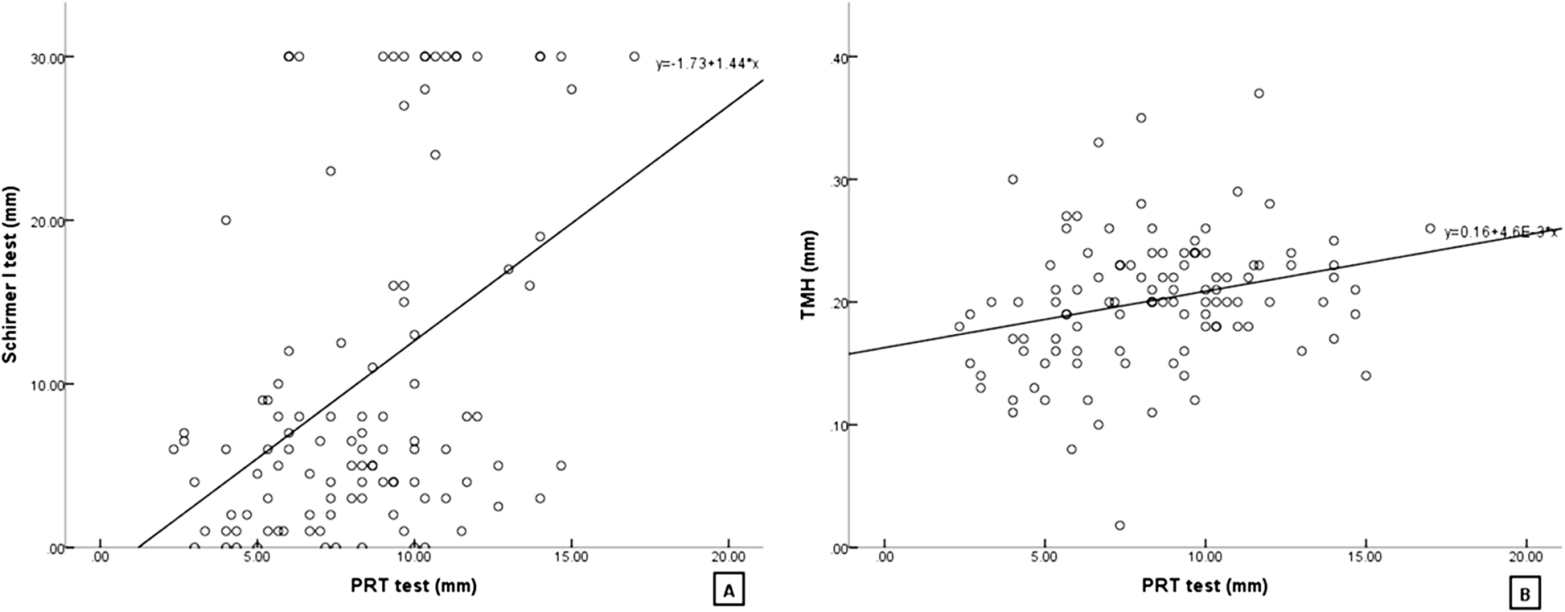
Scatter diagrams showing the correlations between the PRT test and DED parameters. **A** Spearman correlation by rank test between the PRT test and Schirmer I test (*P* < 0.05, ρ = 0. 385). **B** Spearman correlation by rank test between the PRT test and TMH assessment (*P* < 0.05, ρ = 0.306)

The mean values and Spearman rank correlation coefficients for the PRT test and other ocular examination parameters are shown in Table 4 and Fig. 2.

**Table 4:** Correlations among PRT test and the DED^a^ parameters

Parameters	*n*	Mean ± SD^b^/Median(P25,P75)	Spearman’s rho	*P* value
PRT^c^ test-average (mm)	108	8.40 ± 3.14	-	-
Schirmer I test (mm)	108	6.00 (3.00,16.00)	0.385	< 0.001
TMH^d^ (mm)	108	0.201 ± 0.054	0.306	0.001

^a^*DED* dry eye disease, ^b^*SD* standard deviation, ^c^*PRT* phenol red thread, ^d^*TMH* tear meniscus height

Correlations between the PRT test, Schirmer I test and TMH assessment were tested by Spearman rank-order correlation. The Spearman rank correlation coefficient was 0.385 (*P* < 0.001) for the PRT test and Schirmer test, 0.306 (*P* = 0.001) for the PRT test and TMH assessment. As both of *P* values were < 0.05, the PRT test was correlated with the Schirmer I test and TMH assessment. The Spearman rank correlation coefficients were < 0.4 for both tests, indicating relatively weak correlations between the PRT test, Schirmer I test and TMH assessment.

### Accuracy of PRT test diagnosis

**Fig. 3:**
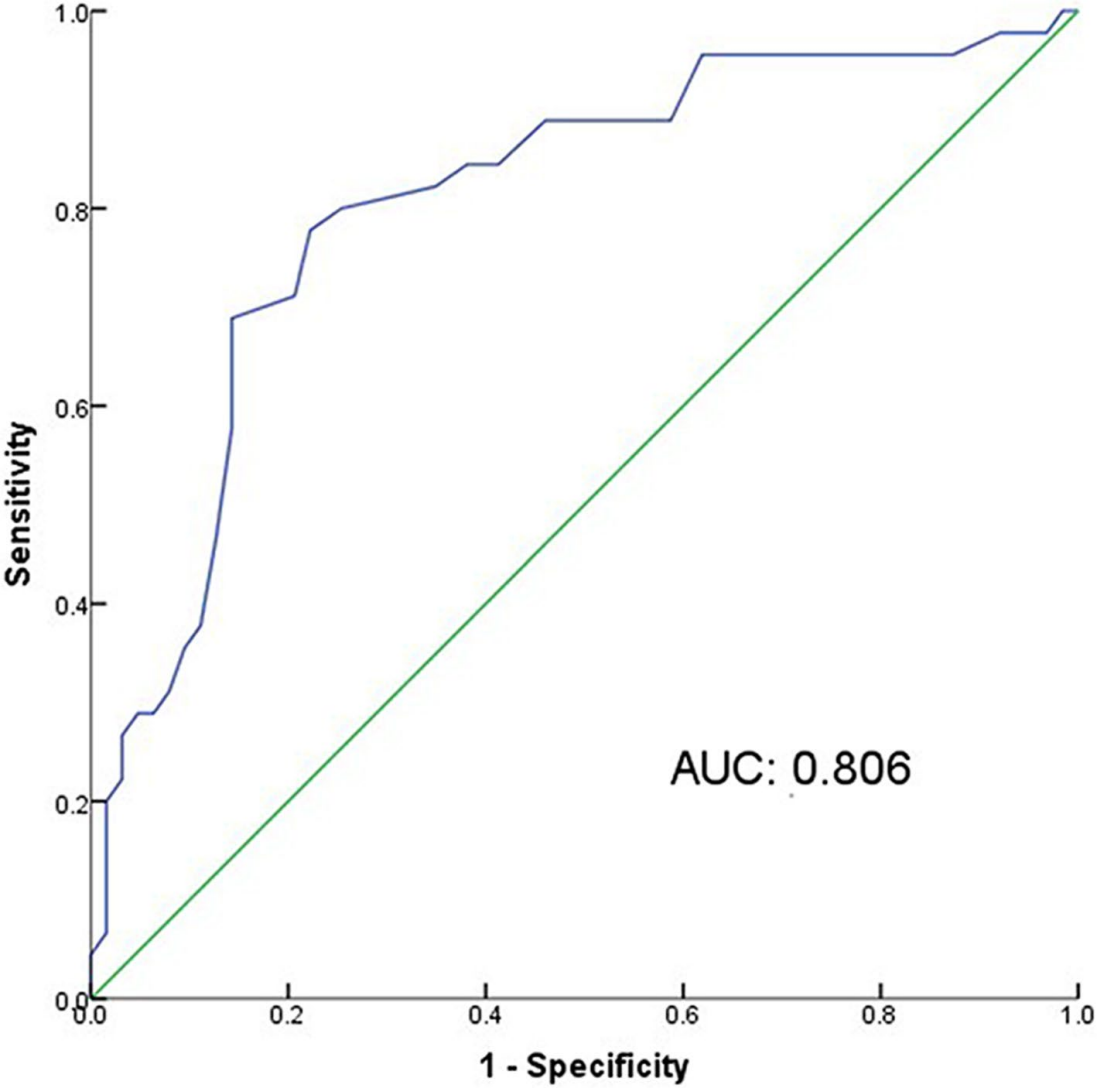
Receiver operating characteristic (ROC) curve for the diagnosis of PRT test in reference to the DED diagnostic criteria. The area under the curve (AUC) value is an important evaluation of the accuracy of a test. (AUC = 0.806)

Receiver operating characteristic (ROC) curve analysis was conducted to determine the accuracy and cutoff point for diagnostic accuracy of the PRT test in reference to the DED diagnostic criteria based on the consensus of DED in China (2020). Graphic and numerical results are shown in Fig. 3 and Table 5, respectively. The area under the curve (AUC) for the PRT test was 0.806 (*P* < 0.001) and the suggested cutoff point was 8.83 mm with a Youden index of 0.556 according to the outcome. As the AUC value was between 0.7 and 0.9, the accuracy of the PRT test was relatively good with regards to the diagnosis of DED.

**Table 5:** The sensitivity, specificity, and Youden index based on each cutoff point of the ROC curve (Fig. 3)

PRT^a^ cutoff points (mm)	Sensitivity	Specificity	*J*^b^
1.3333	1.000	0.000	0.000
2.5000	1.000	0.016	0.016
2.8333	0.978	0.032	0.010
3.1667	0.978	0.063	0.041
3.6667	0.978	0.079	0.057
4.0833	0.956	0.127	0.083
4.2500	0.956	0.143	0.098
4.5000	0.956	0.175	0.130
4.8333	0.956	0.190	0.146
5.0833	0.956	0.222	0.178
5.2500	0.956	0.238	0.194
5.5000	0.956	0.302	0.257
5.7500	0.956	0.365	0.321
5.9167	0.956	0.381	0.337
6.1667	0.889	0.413	0.302
6.5000	0.889	0.444	0.333
6.8333	0.889	0.492	0.381
7.0833	0.889	0.524	0.413
7.2500	0.889	0.540	0.429
7.4167	0.844	0.587	0.432
7.5833	0.844	0.603	0.448
7.8333	0.844	0.619	0.463
8.1667	0.822	0.651	0.473
8.5000	0.800	0.746	0.546
**8.8333**	**0.778**	**0.778**	**0.556**
9.1667	0.711	0.794	0.505
9.5000	0.689	0.857	0.546
9.8333	0.578	0.857	0.435
10.1667	0.467	0.873	0.340
10.5000	0.378	0.889	0.267
10.8333	0.356	0.905	0.260
11.1667	0.311	0.921	0.232
11.4167	0.289	0.937	0.225
11.5833	0.289	0.952	0.241
11.8333	0.267	0.968	0.235
12.3333	0.222	0.968	0.190
12.8333	0.200	0.984	0.184
13.3333	0.178	0.984	0.162
13.8333	0.156	0.984	0.140
14.3333	0.067	0.984	0.051
14.8333	0.044	1.000	0.044
16.0000	0.022	1.000	0.022
18.0000	0.000	1.000	0.000

^a^*PRT* phenol red thread, ^b^*J* Youden index

## Discussion

DED has become an essential public health concern globally and domestically. The Tear Film & Ocular Surface Society Dry Eye Workshop II (TFOS DEWS II) reported that the global prevalence of DED ranged from 5 to 50% and noted that Asian ethnicity is a consistent risk factor [3]. According to recent systematic reviews and meta-analyses, the estimated pooled prevalence of DED in Asia was 20.1% in 2019, the pooled prevalence in China was 17.0% in 2014, and the prevalence of DED by symptoms and signs was 13.55% with a total of 170.09 million people affected in 2010 [19–21]. The consensus on DED in China (2020) defined DED as follows: “Dry eye is a chronic ocular surface disease caused by multiple factors. It is caused by abnormal quality, quantity and dynamics of tears which contributes to unstable tear film or ocular surface microenvironment imbalance, accompanied by an ocular surface inflammatory reaction, tissue damage and nerve abnormalities, resulting in a variety of ocular discomfort symptoms and/or visual dysfunction.” According to this definition of DED, various etiologies are known to contribute to the occurrence of dry eye symptoms and signs. The environmental triggers and exacerbating factors include low ambient humidity, high wind velocity, exposure to airborne particulates and fumes, allergies, nutritional deficiencies, and temperature extremes [22, 23]. Gender and age are also independent risk factors; women are more likely to suffer from dry eyes, and the risk of DED increases with age [3, 24, 25]. Previous studies have reported that women are more predisposed than men to suffer from diseases like thyroid disorders which is also a possible risk factor for DED [26]. Also, diabetes, obesity and high blood pressure are conditions that are closely linked with gender as well as DED, which makes DED more common in women than men [27, 28]. In addition, diseases of the immune system, anatomical and neurological disorders, compromised neural function, meibomian gland dysfunction, graft-versus-host disease, diabetes, hypertension and the use of certain medications may also bring about DED [22, 27, 28]. The highly prevalence of these systematic diseases including hypertension and rheumatic diseases increases the epidemiology of DED worldwide [29, 30].

According to the TFO DEWS II report in 2017, there was no single “gold standard” symptom or sign that completely correlates with DED [4]. Clinicians generally regarded several objective symptoms and various ocular tests as combined diagnostic criteria. The approaches used to diagnose and evaluate DED include the following classifications: questionnaires, corneal and conjunctival vital dye staining, meibomian-gland grading, assessment of tear volume, tear-film stability and tear osmolarity, tear-film interferometry, and the InflammaDry immunoassay [22]. The phenol red thread test is one of the tests used to assess tear volume. The PRT test can be performed in a shorter time period and is more comfortable for the patients than existing tests. Patel et al*.* differentiated aqueous deficient and non-aqueous deficient dry eyes at a 20 mm cutoff point by placing the thread for 120 s, thus resulting in a sensitivity and specificity of 86% and 83%, respectively [17]. In another study, Pult et al*.* placed the thread for 15 s and tested the accuracy of the PRT test at a 10 mm cutoff point; the sensitivity was 25% and the specificity was 93% with an AUC of 0.781 [18]. In this study, the accuracy of the PRT test was also tested in reference to the diagnostic criteria in China and demonstrated relatively good accuracy with an AUC of 0.806. A cutoff point at 8.83 mm was also suggested, at which point the sensitivity and specificity were both 77.8%. Therefore, it is recommended that a cutoff point at 9 mm would be optimal for the clinic with the thread placed for 20 s.

Tests for intra-observer repeatability and inter-examiner reproducibility are key conditions that are used to validate equipment or an inspection method. Intra-observer repeatability, conducted by the same examiner, shows the proportion of variation that can reappear in repeated tests of the same subjects or groups, and can be used to assess the accuracy of a clinical trial or measurement and indicates that the test could become a comparable parameter across studies [31]. Inter-examiner reproducibility is tested by two different examiners using the same raw materials or equipment to carry out the same procedure, and is used to evaluate the generalizability of the approach [32]. Previous studies have shown that the repeatability of the PRT test is good; these findings are in accordance with the present results [33, 34]. We also found that the ICC value for the experimental group was higher than for the control group, thus indicating that the PRT test was more sensitive for DED patients. As for the reproducibility, a previous study from Hong Kong reported that reproducibility was improved after different examiners had been trained in certain manner [35]. The reason why the ICC value for reproducibility was not particularly good in the present study might be because of the personal difference between examiners. For instance, during the examination in present study, the position in which different clinicians placed the thread might vary. Furthermore, the time at which the thread is pulled away might differ between examiners. Therefore, it is recommended that examiners should be trained properly, in a standardized manner, before conducting the PRT test.

The PRT test was initially introduced to replace the Schirmer test; therefore, many researchers investigated the correlations between PRT test and Schirmer test. These studies found that correlations between the two tests were weak and the diagnostic agreement of two tests was poor; these findings are consistent with our present data [33, 36, 37]. Because the Schirmer test is quite invasive, it is possible that results relying on reflex tear secretion may be inconsistent. Some researchers found that the repeatability of the Schirmer test was not good; this may also be a reason for previous inconsistencies [34]. In addition, as the PRT test was designed to measure tear volume, we also evaluated the correlation between the PRT test and TMH assessment and found that the correlation was weak despite the fact that they were positively correlated. The inconformity between present findings and previous reports might be due to the fact that differences between examiners may increase the error created by TMH assessment [9, 34].

In this study, the PRT test was thoroughly evaluated and validated from various aspects in a Chinese population and acquired several lines of evidence that could improve the accuracy of this measurement for clinicians. Firstly, before conducting the test, clinicians should fully inform the subjects of what the test involves; this will eliminate their nervousness and help them to remain calm; the factors that may irritate the ocular surface should also be avoided. Secondly, examiners should be swift and gentle during the procedure, and should be trained appropriately so as to place the thread at the same position in each subject and to pull out the thread and read the outcome as soon as possible. Moreover, the present study found that it was easier for subjects to cooperate with their eyes closed during the procedure; a previous study reported that there was significant differences when the eyes were opened or closed [38]. Subjects should be told to avoid rotating their eyeballs; this will prevent irritation from the thread. In addition, it is more accurate to diagnose DED by combining different kinds of measurement; the PRT test should carried out first to avoid irritation from other tests.

A possible limitation or bias of the present study was the sample size; our limited number of subjects might not be sufficient to represent the results of all DED patients and normal subjects. Apart from that, it was inevitable that individual differences may appear between examiners due to differences in proficiency when conducting the tests.

## Conclusion

The PRT test provided a comfortable, timesaving and less irritating approach for the screening and diagnosis of DED with reliable validation in a Chinese population. We determined satisfactory intra-observer repeatability, moderate inter-examiner reproducibility, and a relatively acceptable diagnostic accuracy, as described in previous studies. The PRT test could be generalized as a practical and efficient measurement for busy clinical services. A study with a large sample size or standardized training of examiners will have broad prospects and significance.

## Data Availability

The datasets used and/or analysed during the current study are available from the corresponding author on reasonable request.

## Abbreviations

AUC: Area under the curve
CV: Coefficient of variance
DED: Dry eye disease
FBUT: Fluorescein tear film breakup time
ICC: Intra-class correlation coefficient
NIBUT: Non-invasive tear breakup time
OSDI: Ocular Surface Disease Index
PRT: Phenol red thread
ROC: Receiver operating characteristic
SD: Standard deviation
TFOS DEWS II: Tear Film & Ocular Surface Society Dry Eye Workshop II
TMH: Tear meniscus height
